# Distribution of host-specific parasites in hybrids of phylogenetically related fish: the effects of genotype frequency and maternal ancestry?

**DOI:** 10.1186/s13071-020-04271-3

**Published:** 2020-08-08

**Authors:** Vadym Krasnovyd, Lukáš Vetešník, Andrea Šimková

**Affiliations:** 1 Department of Botany and Zoology, Faculty of Science, Masaryk University, Kotlářská 2, 611 37 Brno, Czech Republic; 2 Institute of Vertebrate Biology, Czech Academy of Sciences, Květná 8, 603 65 Brno, Czech Republic

**Keywords:** Hybridization, Cyprinid fish, Monogenean infection, Host specificity, Coadaptation

## Abstract

**Background:**

Host specificity is one of the outputs of the coevolution between parasites and their associated hosts. Several scenarios have been proposed to explain the pattern of parasite distribution in parental and hybrid genotypes ranging from hybrid resistance to hybrid susceptibility. We hypothesized that host-parasite co-adaptation limits the infection of host-specific parasites in hybrid genotypes even under the condition of the high frequency of hybrids. The experimental monogenean infection in pure breeds of *Blicca bjoerkna* and *Abramis brama* and cross-breeds (the F1 generation of hybrids) under the condition of similar frequencies of pure and hybrid genotypes was investigated. We also examined the potential effect of the maternal origin of hybrids (potential co-adaptation at the level of mitochondrial genes) on monogenean abundance.

**Methods:**

Pure breeds of two cyprinids and two cross-breeds (one with *B. bjoerkna*, the next with *A. brama* in the maternal positions) were exposed to infection by monogeneans naturally occurring in *B. bjoerkna* and *A. brama*. The experiment was run under similar frequencies of the four breed lines.

**Results:**

We showed similar levels of monogenean infection in *B. bjoerkna* and *A. brama*. However, each species harboured specific monogenean fauna. Hybrids harboured all monogenean species specifically infecting one or the other species. Monogenean infection levels, especially those of *Dactylogyrus* specific to *A. brama*, were lower in hybrids. For the majority of host-specific parasites, there was no effect of the maternal origin of hybrids on monogenean abundance. Asymmetry was found in the distribution of specific parasites in favour of specialists of *B. bjoerkna* in the monogenean communities of hybrids.

**Conclusions:**

Our results indicate that the maternal mtDNA of hybrids is not an important predictor of host-specific monogenean infection, which may suggest that mitochondrial genes are not strongly involved in the coadaptation between monogeneans and their associated hosts. The asymmetry of species-specific parasites suggests similarity between the molecular components of the immune mechanisms in hybrids and *B. bjoerkna*. Our results revealed a difference between the degree of host-parasite coadaptation in specific parasites of *A. brama* and the degree of host-parasite coadaptation in specific parasites of *B. bjoerkna* and their associated hosts.
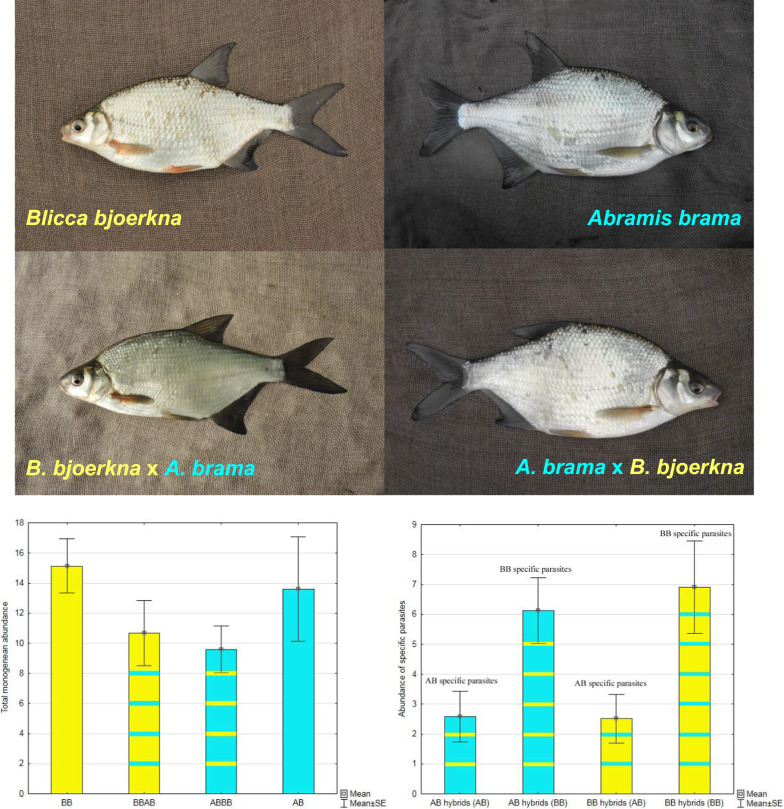

## Background

The evolution of host-parasite relationships is associated with reciprocal genetic co-adaptations between the two interacting partners [1, 2]. Such host-parasite gene-to-gene interactions appeared during co-evolution and long-running arms races between parasite adaptations and host defense mechanisms [3]. The level of parasite infection is regulated by a co-adapted genetic system, which was originally suggested to explain the pattern of parasite distribution in two species of house mice (*Mus musculus* and *M. domesticus*) and their natural hybrids [4, 5]. Host-parasite genetic co-adaptation is more pronounced in host-specific parasites, thus limiting their infection in hybrid genomes [6, 7]. However, several scenarios of parasite infection in hybridizing hosts were proposed to explain the distribution of parasites among hybrids and their parental taxa [8, 9]. Wolinska et al. [9], in their dynamic scenario, hypothesized that hybrid genomes are more parasitized than parental genomes under the condition of the high frequency of hybrids. In addition, it seems that the maternal ancestry of hybrids may also influence the infection level of some parasite species, as was shown for digenean and crustacean species in F1 hybrids of the evolutionarily divergent, and morphologically and ecologically different cyprinid species, the common bream (*Abramis brama*) and roach (*Rutilus rutilus*) [7].

Fish hybridization is a frequent event in nature [10, 11]. Such hybridization very often results from the introduction or invasion of non-native fish species [12–16] caused by direct anthropogenic activities (agriculture, fish farming and waterbodies transformation) or human-mediated disturbances of nature (climate change, interspecies competition or limited living resources). Generally, the presence of F1 hybrids is documented in many wild-living cyprinids even when the frequency of hybrids is rather low [6, 16–22]. Cyprinid hybrids exhibit high larval resistance to environmental disturbances (osmotic and thermal conditions), wider ecological plasticity, greater fasting abilities in comparison to their parents, and limited susceptibility to parasite infection [7, 23–25].

From the evolutionary point of view, hybridization generates novel morphological and genetic variants of organisms. However, novel features of hybrid organisms may affect the compatibilities between hosts and specific parasites. Generally, host-parasite incompatibility (predicted by the resistance hypothesis) or, alternatively, host-parasite compatibility (revealed as phenotypic or genotypic compatibility proposed by the matching hypothesis) result from a wide range of molecular determinants [26]. Moreover, the incompatibility between fish and their ectoparasitic monogeneans might arise from the substrate structure of the fish host (i.e. the different structure of gill filaments of “wrong” hosts) [27, 28].

Differences in monogenean species richness and abundance between hybrids and their parental species were previously shown in fish [6, 7, 16, 29]. Hybrids of cyprinid fish species are infected by a majority of the parasites specific to one or the other parental species in the case of evolutionarily distant cyprinid species [7], or infected by parasites specific to both parental species in the case of evolutionarily closely-related cyprinid species [6]. Interestingly, an asymmetrical distribution of parental species-specific parasites (especially specific monogeneans representing gill ectoparasites) was found in hybrids of *A. brama* and *R. rutilus*. This may indicate the limited inheritance of defense mechanisms from one parental species and suggests different degrees of host-parasite coadaptation between different host-specific parasites and their associated hosts [7, 19].

In the present study, two widely distributed cyprinid species in central European waters exhibiting low evolutionary divergence and high morphological similarities were studied: silver bream (*Blicca bjoerkna*) and common bream (*A. brama*). With respect to these species, the intermediate survival rate of the F1 generation, hybrid fertility, and the capacity to produce high quality gametes have previously been documented [24, 30, 31]. *Blicca bjoerkna* and *A. brama* also exhibit similar ecology. Each of these cyprinid species harbours specific monogenean parasites [32].

The aim of this study was to use breed lines of *B. bjoerkna* and *A. brama* and crossbreed lines representing the F1 generation (i.e. using fish whose immunity was not previously affected by any infection) in order to investigate the susceptibility of the F1 hybrids of two phylogenetically closely-related cyprinid species to the specific monogenean parasites of the parental species and to test whether the maternal ancestry of F1 hybrids (mtDNA) has an effect on the level of monogenean parasite infection.

## Methods

Specimens of *B. bjoerkna* (3 females and 3 males) and *A. brama* (3 females and 3 males) were caught by electrofishing from the River Dyje near the city of Břeclav (48.7566N, 16.8701E; Morava River basin, Czech Republic) and transported alive to the facility of the Institute of Vertebrate Biology, Czech Academy of Sciences. Fish separated according to sex into two well-aerated tanks were stimulated for ovulation/spermiation by carp pituitary (females received 2 doses, 0.3 and 2.7 mg/kg 24 h and 12 h before propagation, respectively; males received 1 mg/kg 24 h before propagation) and by subsequently increasing the water temperature to 22 °C. Oocytes of ovulating females were obtained by the dry method and sperm was sampled according to Linhart et al. [33]. Hatchery water was used for gamete activation. Artificial spawning based on individual pair mating was performed using the following parental combinations: (i) female and male *B. bjoerkna*; (ii) female and male *A. brama*; (iii) female *B. bjoerkna* and male *A. brama*; and (iv) female *A. brama* and male *B. bjoerkna*. As a result of artificial spawning, four lines of offspring were obtained: pure breed *B. bjoerkna*; pure breed *A. brama*; and two crossbreed lines, F1 hybrids with *A. brama* maternal origin (mtDNA) and F1 hybrids with *B. bjoerkna* maternal origin (mtDNA). Four lines of offspring were reared subsequently up to 1-year-old.

The experimental infection was conducted in a well aerated and filtered tank. A total of 15 specimens: 8 of *A. brama* and 7 of *B. bjoerkna* captured from nature (the River Dyje near the city of Břeclav) were placed in the tank with aerated water. To initiate infection, the batches of the *A. brama* and *B. bjoerkna* breed lines and the crossbreed lines were kept in the same tank with the infected specimens of *A. brama* and *B. bjoerkna* caught in nature.

Three weeks after the initiation of the experiment, all fish specimens were sacrificed by severing the spinal cord in accordance with Law 246/1992 of the Czech Republic and subsequently dissected following Ergens & Lom [34]. All parasite specimens were collected, fixed in GAP (glycerine-ammonium picrate) following Malmberg [35], and identified on the basis of the species-specific sclerotized structures of the attachment organ (haptor) and reproductive organs following Gusev [36] using a light microscope (Olympus BX 51; Olympus Life and Material Science Europe GMBH, Hamburg, Germany) with phase-contrast, differential interference contrast, and a digital image analysis system (Stream motion).

Prevalence and intensity of infection were calculated for each parasite species in each of the host lines (*A. brama*, *B. bjoerkna*, F1 hybrids with maternal origin of *A. brama* and F1 hybrids with maternal origin of *B. bjoerkna*). In accordance with Bush et al. [37], prevalence as a percentage of infected fish of a given host line by a given parasite species, and mean intensity of infection (expressed by mean ± standard deviation) as a number of parasite specimens of a given species per infected host, were calculated. The range of intensity of infection was included for each parasite species in each of host lines.

The monogenean species present on *A. brama* and absent on *B. bjoerkna* in this study were classified as *A. brama*-specific parasites. The monogenean species present on *B. bjoerkna* and absent on *A. brama* in this study were classified as *B. bjoerkna*-specific parasites. The delimitation of host specificity for monogenean species primarily originated from the checklist of fish-parasite records from the Czech and Slovak Republics by Moravec [38]. For some monogenean species, one of the two cyprinid species examined in this study represents the most common host species in central European rivers within a wide range of potential host species; for example, *Dactylogyrus sphyrna* is the most common parasite of *B. bjoerkna* within the range of central European cyprinids [38–40]. Such a parasite species was also considered as host specific in the present study.

Kruskal-Wallis H-test followed by multiple comparison tests was applied to test the differences in monogenean abundance among and between fish breeds. As host-specific parasites were not shared between *A. brama* and *B. bjoerkna*, the Kruskal-Wallis H-test followed by multiple comparisons was performed to compare the abundance of specific parasites (i) between *A. brama* and the two hybrid lines, i.e. F1 hybrids with *A. brama* maternal origin and F1 hybrids with *B. bjoerkna* maternal origin, and (ii) between *B. bjoerkna* and the two abovementioned hybrid lines. Asymmetrical infection by host-specific parasites in F1 hybrids with different maternal origins (i.e. differences in the parasite species richness and abundance of *A. brama*-specific parasites and *B. bjoerkna*-specific parasites between F1 hybrids of different maternal origins) was tested using the Wilcoxon signed-rank test. The Mann-Whitney U-test was used to test the effect of the maternal origin of hybrids on the asymmetrical infection by host-specific parasites in F1 hybrids; i.e. the difference between *B. bjoerkna*-specific parasite species richness (or abundance) and *A. brama*-specific parasite species richness (or abundance) was used as a dependent variable in the Mann-Whitney U-test. Statistical analyses were performed using Statistica 13.3 for Windows (TIBCO software Inc., Palo Alto, CA, USA).

## Results

**Fig. 1:**
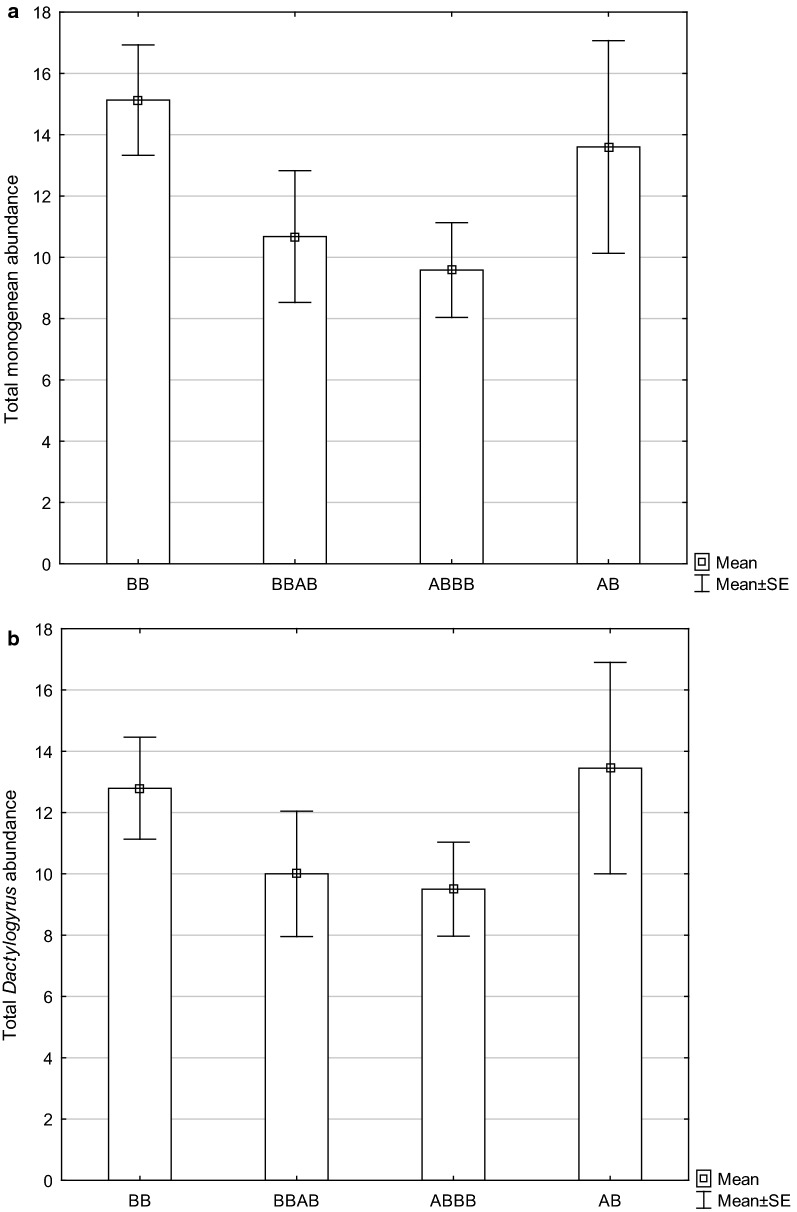
Total monogenean abundance (**a**) and *Dactylogyrus* abundance (**b**) in breed lines of *B. bjoerkna*, *A. brama* and hybrids. *Abbreviations*: AB, *Abramis brama*; BB, *Blicca bjoerkna*; ABBB, hybrids with maternal *A. brama*; BBAB, hybrids with maternal *B. bjoerkna*

The examination of wild living fish specimens revealed 6 monogenean species in *A. brama* and 4 monogenean species in *B. bjoerkna*, all monogeneans were found on the gills (Table 1). No difference in monogenean abundance was found between the two cyprinid species (Mann-Whitney U-test: *U*_(15)_ = 16.5, *Z* = − 1.27, *P* = 0.203). Using fish from artificial breeding, significant differences in total monogenean abundance among *A. brama*, *B. bjoerkna*, and F1 hybrids were found (Kruskal-Wallis H-test: *H*_(2, 126)_ = 6.04, *P* = 0.049). Even when monogenean abundance tended to reach higher values in common bream and silver bream when compared to hybrids, multiple comparisons revealed only a significant difference between F1 hybrids and *B. bjoerkna* (*P* = 0.043) (Fig. 1a). *Dactylogurus* parasites had the dominant position in the monogenean communities of all the investigated fish breed lines (*A. brama*, *B. bjoerkna*, F1 hybrids with *A. brama* maternal origin and F1 hybrids with *B. bjoerkna* maternal origin). The differences in *Dactylogyrus* abundance among *A. brama*, *B. bjoerkna*, and hybrids were not significant (Kruskal-Wallis H-test: *H*_(2, 126)_ = 2.90, *P* = 0.235, Fig. 1b). *Dactylogyrus zandti* and *D. falcatus* were the parasite species with the highest infection levels in the experimentally infected breed line of *A. brama*. Concerning host-specific parasites of *A. brama* present in hybrids, *D. zandti* achieved the highest infection level (Table 2). On *B. bjoerkna*, *D. sphyrna* was the most prevalent parasite, reaching also the highest intensity of infection, followed by the intensities of infection of *D. distinguendus* and *Paradiplozoon bliccae* (Table 2). *Dactylogyrus sphyrna* was also the monogenean species exhibiting the highest infection level (measured by prevalence and intensity of infection) in the F1 hybrids of both maternal origins. Monogenean communities of the *A. brama* breed line consisted of 5 monogenean species; 4 of them host-specific *Dactylogyrus* species, which were also identified in the source specimens of this species (Tables 1, 2). Two species, *Gyrodactylus elegans* and *Diplozoon paradoxum*, were found in source specimens of *A. brama* from nature but were not identified in experimentally infected specimens of this cyprinid species. Monogenean communities of the *B. bjoerkna* breed line consisted of 5 species; 4 of them were found in the sample of source specimens of *B. bjoerkna* collected in nature (Tables 1, 2). The monogenean communities of F1 hybrids exhibited higher monogenean species richness in comparison to those of parental species, i.e. all monogenean species found in breed lines of *A. brama* and *B. bjoerkna* were also present in F1 hybrids.

**Table 1:** Intensity of monogenean infection (MI, mean ± standard deviation), range of intensity of infection (I, minimum-maximum), and prevalence (P, in %) for each monogenean species in *A. brama* and *B. bjoerkna* captured from nature

Parasite species	*Abramis brama* (*n* = 8)			*Blicca bjoerkna* (*n* = 7)		
	MI	I	P	MI	I	P
*Dactylogyrus auriculatus*	1.67 ± 1.16	1–3	38	–	–	–
*Dactylogyrus wunderi*	4.00 ± 2.65	1–6	38	–	–	–
*Dactylogyrus zandti*	9.80 ± 8.90	2–22	63	–	–	–
*Dactylogyrus falcatus*	1.67 ± 0.58	1–2	38	–	–	–
*Dactylogyrus sphyrna*	–	–	–	2.00 ± 1.41	1–3	29
*Dactylogyrus cornu*	–	–	–	4.75 ± 4.35	1–9	57
*Dactylogyrus distinguendus*	–	–	–	1.75 ± 1.5	1–4	57
*Gyrodactylus vimbi*	–	–	–	1.50 ± 0.71	1–2	29
*Gyrodactylus elegans*	1	–	13	–	–	–
*Diplozoon paradoxum*	2	2	63	–	–	–

**Table 2:** Intensity of monogenean infection (MI, mean ± standard deviation), range of intensity of infection (I, minimum-maximum), and prevalence (P, in %) for each monogenean species in pure *A. brama*, pure *B. bjoerkna* and hybrids

Parasite species	*Abramis brama* (*n* = 20)			*Blicca bjoerkna* (*n* = 39)			F1 hybrids with maternal origin of *A. brama* (*n* = 36)			F1 hybrids with maternal origin of *B. bjoerkna* (*n* = 31)		
	MI	I	P	MI	I	P	MI	I	P	MI	I	P
*Dactylogyrus auriculatus*	1	1	10	–	–	–	1	1	6	1	1	10
*Dactylogyrus wunderi*	4.00 ± 3.00	1–10	55	–	–	–	1	1	8	1	1	10
*Dactylogyrus zandti*	7.93 ± 8.93	1–33	70	–	–	–	7.36 ± 8.24	1–24	31	4.90 ± 5.59	1–19	32
*Dactylogyrus falcatus*	6.13 ± 5.50	1–18	75	–	–	–	2.78 ± 2.77	1–8	25	2.30 ± 1.25	1–4	32
*Dactylogyrus sphyrna*	–	–	–	11.83 ± 9.34	1–31	79	7.08 ± 7.02	1–27	69	9.80 ± 10.38	1–41	48
*Dactylogyrus cornu*	–	–	–	3.53 ± 2.61	1–9	38	1.73 ± 1.27	1–4	31	2.75 ± 2.49	1–8	26
*Dactylogyrus distinguendus*	–	–	–	5.77 ± 5.78	1–19	33	3.36 ± 2.95	1–10	25	3.73 ± 4.05	1–15	35
*Gyrodactylus vimbi*	1	1	15	1	1	8	2	–	3	3.40 ± 4.28	1–11	16
*Paradiplozoon bliccae*	–	–	–	5.50 ± 4.13	1–13	41	1	–	3	1.33 ± 0.58	1–2	10

Concerning *Dactylogyrus* spp. specific to *A. brama*, a significant difference (Kruskal-Wallis H-test: *H*_(2, 87)_ = 17.09, *P* = 0.002, multiple comparisons between *A. brama* and F1 hybrids with *A. brama* maternal origin: *P* < 0.001 and between *A. brama* and F1 hybrids with *B. bjoerkna* material origin: *P* = 0.002) was revealed for total *Dactylogyrus* abundance. The same difference was found for the abundance of 3 *Dactylogyrus* species, i.e. *D. falcatus* (Kruskal-Wallis H-test: *H*_(2, 87)_ = 18.82, *P* < 0.001, multiple comparisons: *P* < 0.001 and *P* = 0.006, respectively), *D. wunderi* (Kruskal-Wallis H-test: *H*_(2, 87)_ = 24.38, *P* < 0.001, multiple comparisons: *P* = 0.005 and *P* = 0.008, respectively), and *D. zandti* (Kruskal-Wallis H-test: *H*_(2, 87)_ = 9.84, *P* = 0.007, multiple comparisons: *P* = 0.033 and *P* = 0.042, respectively). However, no differences in total *Dactylogyrus* abundance or the abundance of individual *Dactylogyrus* species between the 2 lines of hybrids (*P* > 0.05) were found. No difference in the abundance of least numerous *D. auriculatus* between *A. brama* and hybrids (Kruskal-Wallis H-test: *H*_(2, 87)_ = 0.51, *P* = 0.775) was found.

Concerning *Dactylogyrus* specific to *B. bjoerkna*, the Kruskal-Wallis H-test followed by multiple comparisons revealed a weakly significant difference in the abundance of only *D. sphyrna* between *B. bjoerkna* and hybrids with *B. bjoerkna* maternal origin (Kruskal-Wallis H-test: *H*_(2, 106)_ = 6.99, *P* = 0.03, multiple comparison: *P* = 0.046). Concerning *P. bliccae*, a significant difference was found between *B. bjoerkna* and hybrids with *A. brama* maternal origin (Kruskal-Wallis H-test: *H*_(2, 106)_ = 21.58, *P* < 0.001, multiple comparison: *P* = 0.009). The difference between *B. bjoerkna* and hybrids with *B. bjoerkna* maternal origin was not significant (multiple comparison: *P* = 0.052).

*Gyrodactylus vimbi* was the only monogenean species infecting both pure breed lines and both lines of hybrids (Table 2). No significant differences in the abundance of *G. vimbi* were found among the four groups of fish (Kruskal-Wallis H-test: *H*_(3, 126)_ = 4.33, *P* = 0.228).

**Fig. 2:**
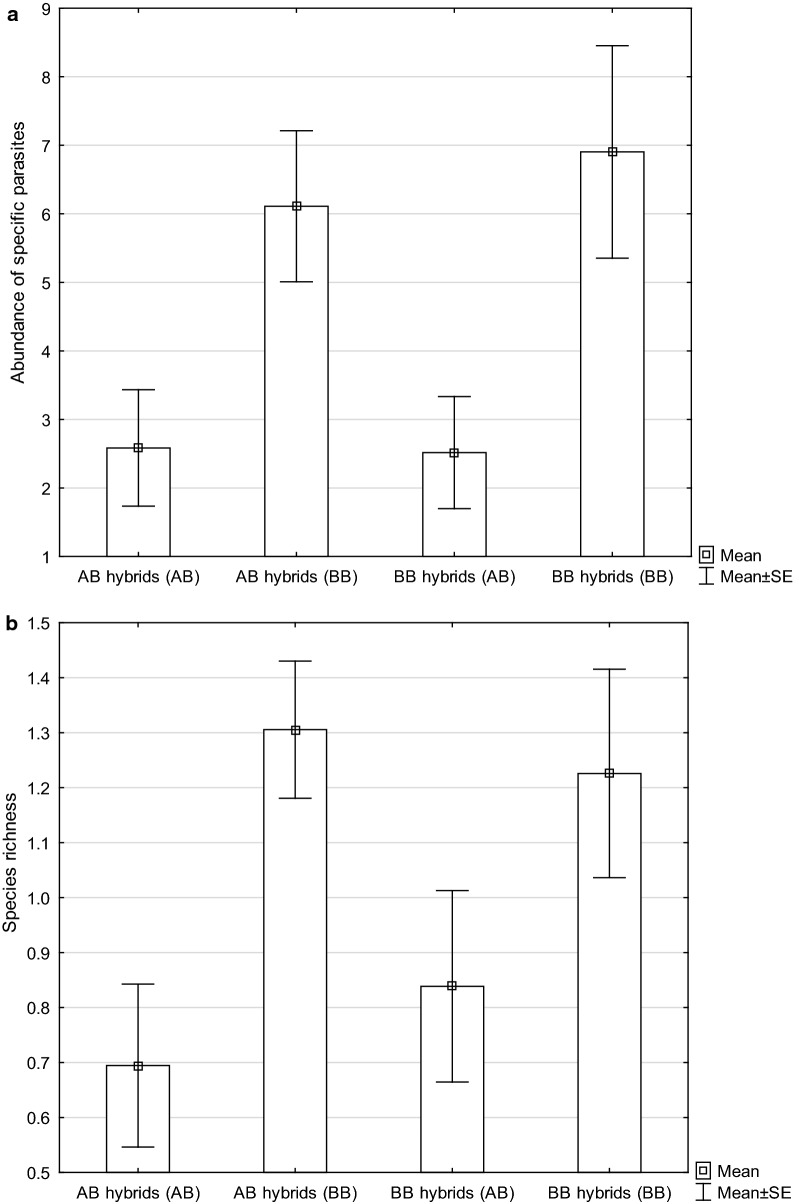
Asymmetrical distribution of parental-specific parasites in hybrids with different maternal origins. Abundance (**a**) and species richness (**b**) of *A. brama*-specific parasites (indicated as AB in parentheses on the x-axis) and *B. bjoerkna* -specific parasites (indicated as BB in parentheses on the x-axis) in hybrids with *A. brama* maternal origin (AB hybrids) and hybrids with *B. bjoerkna* maternal origin (BB hybrids)

The Wilcoxon signed-rank test revealed asymmetry in the abundance of host-specific monogeneans in favor of parasites specific to *B. bjoerkna* (Fig. 2a). The abundance of *B. bjoerkna*-specific parasites was higher than the abundance of *A. brama*-specific parasites in the hybrid line with *B. bjoerkna* maternal origin (*Z* = 2.99, *P* = 0.003) and the hybrid line with *A. brama* maternal origin (*Z* = 2.98, *P* = 0.003). The species richness of *B. bjoerkna*-specific parasites was higher than the species richness of *A. brama*-specific parasites in the hybrid line with *B. bjoerkna* maternal origin (*P* = 0.049) and the hybrid line with *A. brama* maternal origin (*P* = 0.003) (Fig. 2b). The Mann-Whitney U-test test showed no effect of the maternal origin of hybrids on the difference in monogenean species richness (*U*_(67)_ = 462.00, *Z* = 1.20, *P* = 0.230) or abundance (*U*_(67)_ = 552.00, *Z* = 0.07, *P* = 0.945) between *A. brama*-specific and *B. bjoerkna*-specific parasites.

## Discussion

The presented study focused on the distribution of host-specific parasites (gill monogeneans) in the F1 hybrids of cyprinid species with low evolutionary divergence, *A. brama* and *B. bjoerkna*. Although there is phylogenetic proximity between these cyprinid species [41], they harbor different host-specific monogenean species, especially representatives of *Dactylogyrus*. The majority of host-specific *Dactylogyrus* in European cyprinid hosts evolved by intra-host speciation (speciation within host species) [39], i.e. *D. zandti* and *D. wunderi* specific to *A. brama* evolved by intra-host speciation within *A. brama*, and *D. distinguendus*, *D. cornu* and *D. cornoides* specific to *B. bjoerkna* evolved by intra-host speciation within *B. bjoerkna*. Both *A. brama* and *B. bjoerkna* are also parasitized by *Dactylogyrus* species, representatives of other phylogenetic lineages unrelated to those originating from intra-host speciation (more specifically, *D. auriculatus* of *A. brama* and *D. sphyrna* of *B. bjoerkna* likely originate from host switching). Despite the phylogenetic proximity of *A. brama* and *B. bjoerkna*, their *Dactylogyrus* species (or lineages) evolved independently (i.e. *Dactylogyrus* species host-specific to *A. brama* and *Dactylogyrus* species host-specific to *B. bjoerkna* are not phylogenetically closely related [39]).

The present work is the first experimental study investigating the distribution of host-specific monogeneans in F1 hybrids of cyprinids under the following conditions: (i) the experiment was run using similar proportions of parental and hybrid genotypes; and (ii) the immunity of breed and crossbreed lines was not affected by any previous infection prior to experimental infection by monogeneans (in contrast to the previous studies of Šimková et al. [6] and Krasnovyd et al. [7] investigating natural parasite infection of cyprinids).

We applied co-habitation design of experiment when specimens of breed and crossbreed lines were placed together with wild *A. brama* and *B. bjoerkna*. Almost all monogenean parasites reported in *A. brama* or *B. bjoerkna* from nature were also found in experimental specimens. The absence of two monogenean species in experimental specimens is a result of low parasite abundance in wild fish specimens (for *G. elegans*) or failed parasite reproduction under our experimental conditions (for *D. paradoxum*). In contrast, *P. bliccae*, host-specific parasite of *B. bjoerkna*, was not identified in our low sample size of dissected wild fish specimens, but this diplozoid species was evidently formerly present in cohabited wild fish specimens and was successfully reproducing in experimental *B. bjoerkna* and both lines of hybrids.

We found that the total abundance of monogeneans (7 of 9 monogenean species belonged to *Dactylogyrus*) tended to be higher in parental species when compared to hybrids (even if the difference with respect to total monogenean abundance was significant only between *B. bjoerkna* and hybrids). However, for the majority of host-specific parasites (especially for *Dactylogyrus* specific to *A. brama*), we found higher abundance in a corresponding pure species when compared to parasite abundance in hybrids. Fritz et al. [42] proposed four static scenarios explaining the pattern of parasite distribution in parental species and their hybrids. The dominance scenario predicts similar resistance between hybrids and one of the parental taxa; thus, parasite infection levels in hybrids and one parental taxon are similar. The hybrid resistance scenario predicts the superior resistance of hybrids when compared to parental taxa. Therefore, the parasite infection level is lower in hybrids when compared to each of the parentals. The additive scenario predicts a difference in parasite abundance between parental taxa; however, hybrid resistance does not differ from the average resistance of parental taxa. The susceptibility scenario is based on the prediction of the lower resistance of hybrid individuals when compared to parental taxa. In this case, the parasite infection level is higher in hybrids when compared to each of the parental taxa.

Wolinska et al. [9] proposed a dynamic scenario based on the frequencies of common and rare genotypes, i.e. frequency-based selection, to explain the temporal dynamics in parasite infection in hybridizing systems of hosts. Such a dynamic scenario incorporates all the phases described as four static scenarios by Fritz et al. [42]. Krasnovyd et al. [7] investigated the pattern of parasite distribution in two non-congeneric cyprinids with high evolutionary divergence, *A. brama* and *R. rutilus*, and their F1 hybrids. They showed that hybrids exhibit lower parasite intensities (especially a lower intensity of infection by host-specific parasites) than parental species, this pattern of infection was revealed in a different time and space. The same finding was demonstrated by Šimková et al. [6] using two phylogenetically closely-related cyprinids, i.e. *Cyprinus carpio* and *Carassius gibelio*, that share some *Dactylogyrus* species exhibiting so-called phylogenetic specificity, i.e. parasite species restricted to phylogenetically closely-related host species (for details see Šimková et al. [40]). Krasnovyd et al. [7] suggested that the absence of a pattern proposed by the dynamic scenario of Wolinska et al. [9] is related to the constantly low frequency of hybrid genotypes in nature. In fact, a very low frequency of hybrids was reported in both previous studies [6, 7] investigating the parasite distribution in hybridizing cyprinids. In contrast, Dupont & Crivelli [43] reported a high level of parasite infection in hybridizing *Rutilus rubilio* and *Alburnus alburnus* from Lake Mikri Prespa in northern Greece, where an extremely high rate of hybridization was reported. In the present study, we set our experimental conditions by using similar proportions of *A. brama*, *B. bjoerkna* and hybrid genotypes. Our observation of the trend of a higher overall abundance of monogeneans and of significantly higher abundances of the majority of host-specific parasites in corresponding associated host species when compared to hybrids may suggest, in contrast to the hypothesis of Wolinska et al. [9], that the frequency of host genotypes (pure breeds *versus* crossbreeds) does not play a significant role in host-specific parasite selection.

The hybrid bridge hypothesis [44, 45] suggests that hybrid individuals act as a bridge between parental species transferring their parasites. However, concerning strictly specific parasites, reciprocal co-adaptation between hosts and associated parasite genotypes is hypothesized [46]. This may indicate that fully adapted parasites cannot be transmitted through the “hybrid bridge” from one parental species to another. Sage et al. [4] documented a high level of parasite infection in two house mice species (*Mus musculus* and *M. domesticus*) and their natural hybrids, and suggested that a broken system of host-parasite genetic coadaptation might explain their finding. In the present study, each parental species harbored unique monogenean fauna (except for *G. vimbi*, which was shared by both species), and interspecific hybrids resulting from artificial breeding harbored all parasites specific to *A. brama* and *B. bjoerkna*, mostly at lower intensities of infection when compared to parental species. This pattern of distribution of parental species-specific parasite in hybrids likely results from a lack of genetic co-adaptation between the host-specific parasite and associated host genome (here the genome of *A. brama* or genome of *B. bjoerkna*) as suggested by Krasnovyd et al. [7]. Some monogenean species expressed similar infection levels in parental species and F1 hybrids; these were the three host-specific *Dactylogyrus* species: *D. distinguendus*; *D. auriculatus*; *D. cornu*; and the generalist *G. vimbi*. However, the last three species were found in very low intensities in parental species and hybrids. To test the hybrid bridge hypothesis, other experiments with hybridizing systems of *A. brama* and *B. bjoerkna* (or other fish species with host-specific parasites) will need to be performed in the future, these focusing on the different rate of genetic introgression between two species (the F1 generation, back-crosses, and the F2 generation of hybrids) and the potential to transfer host-specific parasites. However, the hybridization of *A. brama* and *B. bjoerkna* does not represent a serious threat to the genetic integrity of these species (because of the low frequency of hybrids in nature) and presents only a minimal risk with respect to transferring host-specific parasites (because of host-parasite co-adaptation).

In the present study, we also examined the potential effect of mtDNA on the presence of host-specific parasites. Cyto-nuclear incompatibility, i.e. incompatibilities between the alleles of mitochondrial and nuclear genomes resulting from hybridization, may cause hybrid breakdown even in the F1 generation of hybrids [47]. Chou & Leu [47] stated that some diseases related to mitochondrial DNA are pathogenic only in certain nuclear backgrounds of hosts. However, in our study, no obvious evidence of an mtDNA effect on the level of infection of host-specific monogeneans in hybrids was found. It seems that maternal origin, i.e. represented by mitochondrial genes, is not primarily involved in the association between fish hosts and their specific parasites. *Paradiplozoon bliccae*, a strict specialist of *B. bjoerkna*, was the only parasite species exhibiting a significant difference in abundance between silver bream and hybrids with common bream maternal origin. Due to the low parasite intensity of infection, this finding cannot clearly support the effect of mtDNA on the presence and/or level of parasite infection. However, even with no statistical support, our data indicate a higher intensity of infection by *B. bjoerkna*-specific parasites in hybrid specimens with *B. bjoerkna* maternal position.

Šimková et al. [19] suggested host-parasite co-evolutionary associations as a major factor limiting the distribution of host-specific *Dactylogyrus* parasites in pure and hybrid specimens across hybrid zones. In a study focusing on the temporal and spatial variation of parasite infection in a hybridizing system of cyprinid species with high evolutionary divergence, Krasnovyd et al. [7] demonstrated asymmetry in the proportions of parental species-specific parasites in the parasite communities of hybrids. They suggested that the different inheritances of immune protective mechanisms from two parental species or alternatively the different rates of co-adaptation between specific parasites and their associated hosts may explain this asymmetry. In their study, the parasite communities of hybrids were shifted toward a higher proportion of host-specific parasites (especially monogeneans) of *R. rutilus*, which was the species achieving higher monogenean diversity but lower parasite (and also monogenean) abundance when compared to *A. brama*. In our study, we reported asymmetry in the monogenean communities of hybrids toward a higher abundance of *B. bjoerkna*-specific parasites. However, source fish caught in nature as well as breed lines of *A. brama* and *B. bjoerkna* exhibited no differences in monogenean intensity of infection, and *A. brama* even exhibited slightly higher total monogenean species richness.

## Conclusions

Our results confirm that the presence of host-specific parasites is affected by hybridization. We showed that host-specific monogeneans reached higher levels of infection in pure species when compared to their reciprocal intergeneric hybrids under the condition of there being similar proportions of pure species genotypes and hybrid genotypes, which again supports the static scenario of hybrid resistance. This was more strongly evidenced for the abundant *Dactylogyrus* specific to *A. brama*. Our findings contradict Wollinska et al. [9], who hypothesized that host genotypes present in higher frequency are the target of parasite selection, i.e. if hybrids represent the frequent genotype (i.e. common genotype) they are more susceptible to parasite infection. However, we suggest that the low intensity of infection by host-specific monogeneans in cyprinid hybrids results from a lack of genetic co-adaptation. Monogenean communities exhibited a shift toward a higher proportion of host-specific parasites of *B. bjoerkna* potentially resulting from different inheritances of immune protective mechanisms from both parental species. The maternal origin of hybrids has no principal role in determining the presence of host-specific parasites, which seems to suggest that mitochondrial genes are not primarily involved in co-evolutionary associations between cyprinid hosts and specific monogenean parasites. We highlight the need for genomic studies to identify the genes involved in reciprocal genetic co-adaptations of host-specific parasites and their associated hosts.

## Data Availability

Data upon which the conclusions are based are provided within the article.

## Abbreviations

F1 hybrids: first filial generation of offspring produced by intercrossing of different parental species
F2 hybrids: second filial generation of offspring produced by intercrossing of F1 individuals
mtDNA: mitochondrial DNA which is located in mitochondria within eukaryotic cells
